# Reaction of an Ion and a Free Radical near 0 K: He^+^ + NO → He + N^+^ + O

**DOI:** 10.1021/acs.jpca.2c08221

**Published:** 2023-02-08

**Authors:** Valentina Zhelyazkova, Fernanda B. V. Martins, Serena Schilling, Frédéric Merkt

**Affiliations:** Laboratory of Physical Chemistry, ETH Zürich, CH-8093 Zürich, Switzerland

## Abstract

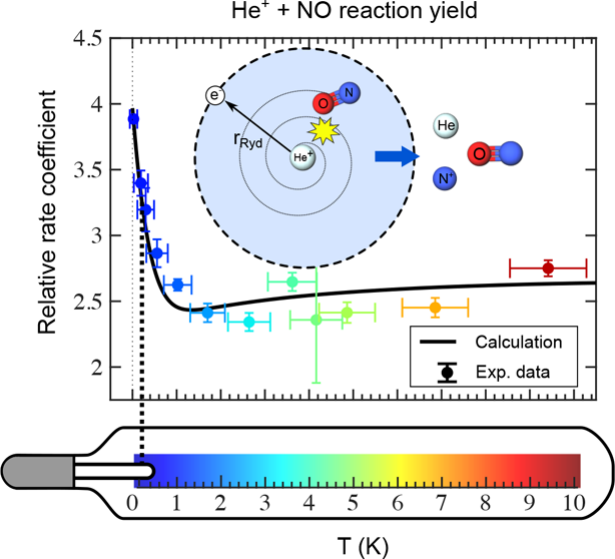

The reactions between ions and free radicals are among the fastest chemical reactions. They are predicted to proceed with large rates, even near 0 K, but so far, this prediction has not been verified experimentally. We report on measurements of the rate coefficient of the reaction between the ion He^+^ and the free radical NO at collision energies in the range between 0 and ∼ *k*_B_·10 K. To avoid heating of the ions by stray electric fields, the reaction is observed within the large orbit of a Rydberg electron of principal quantum number *n* ≥ 30, which shields the ion from external electric fields without affecting the reaction. Low collision energies are reached by merging a supersonic beam of He Rydberg atoms with a supersonic beam of NO molecules and adjusting their relative velocity using a chip-based Rydberg–Stark decelerator and deflector. We observe a strong enhancement of the reaction rate at collision energies below ∼*k*_B_·2 K. This enhancement is interpreted on the basis of adiabatic-channel capture-rate calculations as arising from the near-degenerate rotational levels of opposite parity resulting from the Λ-doubling in the X ^2^Π_1/2_ ground state of NO. With these new results, we examine the reliability of broadly used approximate analytic expressions for the thermal rate constants of ion–molecule reactions at low temperatures.

## 1 Introduction

Free radicals and ions are typically highly reactive species. Many reactions involving free radicals or ions are therefore strongly exothermic, with small barriers or no barriers at all separating reactants and products. Their rate coefficients can thus be large, even near 0 K, where the rates of most reactions become vanishingly small because of finite potential barriers along the reaction coordinates. Ion–radical reactions, i.e., chemical reactions involving *both* ions and free radicals, are particularly likely to be exothermic, barrier-free reactions. Consequently, they are ideal systems to study fundamental aspects of low-temperature chemical reactivity. At very low temperatures, the wave nature of the reactants is expected to become important, and alignment and orientation effects of the reactants in the anisotropic long-range interaction potentials may lead to pronounced stereodynamic effects. Such effects are of fundamental interest in chemistry.

Ion–radical reactions are also expected to play an important role in the chemistry of cold, low-density environments such as interstellar molecular clouds. In such environments, the only gas-phase chemical reactions that take place are exothermic two-body processes without activation energy.^1−4^ Their rate coefficients are needed to model the chemical reaction networks that lead to the formation of complex molecules in space.^4,5^

Ion–radical reactions are difficult to study experimentally because the high reactivity of ions and radicals prevents the buildup of large concentrations; consequently, their yields remain low despite the large values of the rate coefficients. Low-temperature studies (i.e., below ∼10 K) are additionally complicated by the need to cool the ions and the radicals and to reliably control and monitor their kinetic temperature and the distribution of populated rovibrational levels. Very few low-temperature experimental investigations of reactions between ions and free radicals can be found in the literature, and almost all involve laser-cooled atomic free radicals in electronically excited states, see, e.g., refs (6−8). The only experimental study of reactions between ions and molecular radicals near 0 K concerns the reactions C^+^ + NO and C^+^ + O_2_, for which Mazely and Smith measured rate coefficients at 0.6 K in a supersonic-flow apparatus.^9^ However, no details were provided on the measures taken to avoid stray electric fields in the experiments, which could have significantly heated the ions.

In the absence of experimental data, approximate capture models are used to estimate the rates of barrier-free exothermic reactions. The simplest capture model for ion–molecule reactions, originally formulated by Langevin,^10^ considers only the long-range charge−induced-dipole interaction between the ion and the polarizable rotating neutral molecule, with potential scaling with the intermolecular distance *R* as −α′*e*^2^/(8*πϵ*_0_*R*^4^) (α′ is the polarizability volume of the neutral molecule). This model assumes that downhill reactions take place with 100% probability when the reactants come in close contact, i.e., when the collision energy is larger than the potential barriers caused by the centrifugal repulsion term *L*^2^/(2*μR*^2^) (*L* and μ are the angular momentum and the reduced mass of the colliding sepcies, respectively) in the effective long-range interaction potential
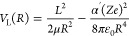
1In this case, the rate constant *k*_L_ does not depend on the temperature (nor on the collision energy) and is given by
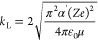
2

When the neutral molecule has a permanent dipole moment or a quadrupole moment, additional, anisotropic terms arise in the long-range interaction potential. At low temperatures, these terms strongly affect the rate coefficients, which can significantly deviate from *k*_L_ and become strongly dependent on the rotational state of the neutral molecule. In this case, approximations are often used to include the effects of the charge-dipole long-range interactions, such as the locked-dipole approximation^11^ or the average-dipole-orientation approximation,^12^ as recently reviewed by Tsikritea et al.^13^ Alternatively, rate coefficients determined at elevated temperatures can be extrapolated down to low temperatures.^14^ More elaborate theoretical treatments are also used to calculate state-specific reaction rate coefficients, such as statistical adiabatic-channel capture models for ion–molecule reactions.^15,16^ These models have been recently tested and validated down to below 1 K for reactions involving ions and closed-shell molecules having permanent electric dipole, quadrupole, and octupole moments.^17−20^

Several adiabatic-channel calculations of capture rates for ion–radical reactions have also been reported which treat the ion as a point charge (see, e.g., refs (21−24)). Compared to reactions between ions and full-shell neutral molecules, reactions involving neutral molecular free radicals pose the challenge of having to include the effects of near-degenerate fine- and hyperfine-structure components of the rotational states. In the case of the X ^2^Π_Ω_ (Ω = 1/2, 3/2) ground state of NO, for example, each rotational level consists of a near-degenerate pair of states of opposite parity resulting from Λ-doubling. In the electric field emanating from the colliding ion, these levels undergo a near-linear Stark effect at long-range, which strongly affects the long-range interaction potentials and the capture rates. In their theoretical studies, Wickham et al.^21^ and Dashevskaya et al.^25^ have predicted higher rate coefficients than those measured by Mazely and Smith,^9^ and Dashevskaya et al. argued that the discrepancy might arise from a rotational temperature of about 20 K of the NO molecules in the supersonic jet. Later, Auzinsh et al. investigated the role of nonadiabatic transitions at very low temperatures in the C^+^ + NO reaction system.^23,24^

More generally, Wickham et al.^21^ wrote already in 1992: “Experimental study in this area is now required. Particular emphasis should be placed on the variation of the rate coefficient with temperature close to 0 K. With very reactive species at such low temperatures, rate coefficient measurement will be difficult but the predictions made here suggest they will be worthwhile.” 30 years later, the situation on the experimental side has just started to improve significantly. Several experimental tools and techniques have become available that are starting to yield insights on the reactions of ions and molecular free radicals at low temperatures. These tools and techniques include cold-ion traps and Coulomb crystals for the generation of (ultra)cold ion samples,^26−29^ as well as buffer-gas cooling,^30,31^ laser cooling,^32,33^ and Zeeman,^34^ Stark,^35^ and Rydberg–Stark^36,37^ deceleration for the generation of (ultra)cold samples of neutral molecules, with Zeeman deceleration being particularly attractive for free radicals.^38−42^ Merged-beams techniques involving short-pulse supersonic jets have also opened ways of studying chemical reactions over broad ranges of collision energies, down to 0 K, with very high energy resolution.^43−47^

To study ion–molecule reactions in the range of collision energies between 0 and ∼*k*_B_·50 K, we have developed a technique exploiting a curved chip-based surface-electrode Rydberg–Stark decelerator to merge beams of Rydberg atoms or molecules with beams of ground-state neutral molecules.^47^ The Rydberg electron, which moves on a distant orbit around the ion core, does not influence the ion–molecule reaction taking place within its orbit but protects the ion core from being heated up by stray fields. In this article, we present a study of the reaction He^+^ + NO → He + N^+^ + O with this technique and focus on the region between 0 and 10 K, where the effects of the open-shell ^2^Π nature of NO on the reaction rate become dominant.

## 2 Experimental Method and Setup

We study the He^+^ + NO reaction at low collision energies in a merged-beam setup. To reach energies near ∼0 K, He^+^ is replaced by a helium atom in a Rydberg state of high principal quantum number (*n* ≥ 30). The Rydberg electron does not affect the reaction between the ion core and the molecule but makes it insensitive to stray-electric-field heating.^18,47−50^

**Figure 1:**
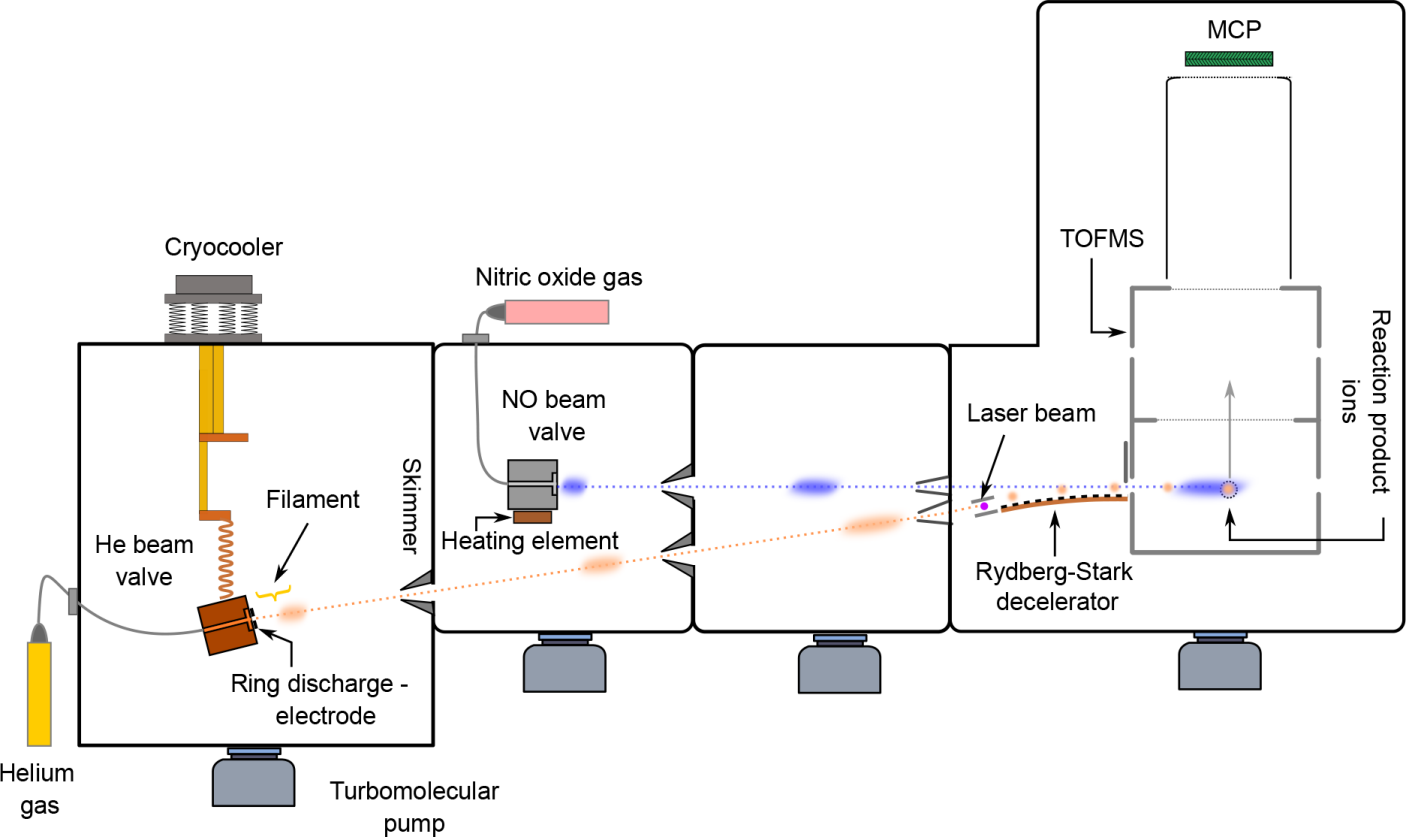
Schematic of the experimental setup.

The experimental setup is described in detail in refs (17−19, 50), and a schematic is presented in Figure 1. Briefly, we use two home-built short-pulse valves (pulse duration ∼20 μs, repetition rate 25 Hz) to produce a supersonic beam of He atoms and a supersonic beam of pure NO molecules. The He valve is cooled with a two-stage pulse-tube cooler and temperature-stabilized to 66 ± 0.1 K, resulting in a mean forward velocity of the helium beam of ∼860 m/s. A pulsed electric discharge at the valve orifice populates the metastable (1s)(2s) ^3^S_1_ state of helium.^51^ Approximately 1 m downstream from the valve, the helium beam is intersected by a pulsed laser beam (wavelength ∼260 nm) which drives a one-photon transition in the presence of an electric field from the metastable (1s)(2s) ^3^S_1_ state to a selected low-field-seeking Rydberg–Stark state (*n*, *k* = *n* – 9, *m* = 0), with *n* in the range between 30 and 45 [*m* is the magnetic quantum number and *k* labels the Rydberg–Stark state and can take on the values: *k* = −(*n* – |*m*| – 1):2:(*n* – |*m*| – 1)].^52^ After excitation, the helium Rydberg atoms [referred to as He(*n*)] are merged with the NO supersonic beam, also generated by a short-pulse valve, using a 50-electrode chip-based Rydberg–Stark deflector and decelerator.^17,18,53^ The deflector is also used to set the mean final velocity *v*_Ryd_ of the Rydberg atoms. The trap volume is small, and the He(*n*) cloud remains compact and has a diameter of about 1–2 mm in the reaction region. The valve used for the NO supersonic beam is temperature-stabilized to 340 ± 0.5 K, resulting in a mean forward velocity *v*_NO_ of ∼870 m/s measured with two fast-ionization gauges placed beyond the reaction zone.^18^ Only the lowest two rotational levels of the ^2^Π_1/2_ ground state are significantly populated at the rotational temperature of ∼4 K of the supersonic expansion.

After the He(*n*) atoms are merged with the NO molecules, they enter a Wiley–McLaren time-of-flight (TOF) mass spectrometer, where all ions in the reaction volume are extracted toward a microchannel-plate (MCP) detector. By the time the NO molecules reach the reaction region, the pulse has dispersed significantly in the longitudinal direction, so that only a very narrow velocity class of the NO molecules overlaps with the He(*n*) cloud. The width of the distribution of relative velocities is thus determined almost exclusively by the velocity distribution of the He(*n*) beam. The collision energy is given by , where μ is the reduced mass of the collision partners. We vary *E*_coll_ by changing *v*_Ryd_ with the surface-electrode deflector, while keeping the velocity of the NO beam fixed. The final velocity of the He(*n*) beam can be set to any value in the range between ∼650 and ∼1100 m/s, corresponding to collision energies between 0 and ∼*k*_B_·10 K. The experimental collision-energy resolution, Δ*E*_coll_, is given by^17^
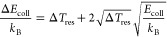
3where Δ*E*_coll_/*k*_B_ is the full width at half-maximum of a Gaussian function. The resolution therefore deteriorates with increasing collision energy. At *E*_coll_ = 0, Δ*E*_coll_ is primarily determined by the velocity distribution of the Rydberg atoms at the end of the surface deflector, as just mentioned, and is characterized by an effective temperature Δ*T*_res_, which is about 100 mK for the experiments presented here (see below).

The ionic reaction products are extracted toward a microchannel-plate detector by applying a pulsed electric field after the He(*n*) Rydberg atoms have reached the center of the reaction zone. The masses of the product ions are determined from the ion flight times to the detector. By monitoring the product-ion yield as a function of the collision energy, we obtain the collision-energy dependence of the reaction rate coefficient. Because we do not measure the absolute densities of the NO molecules and He(*n*) Rydberg atoms in the reaction region, our experiments do not provide absolute rate coefficients or cross sections, but their collision-energy dependence.

## 3 Experimental Results

**Figure 2:**
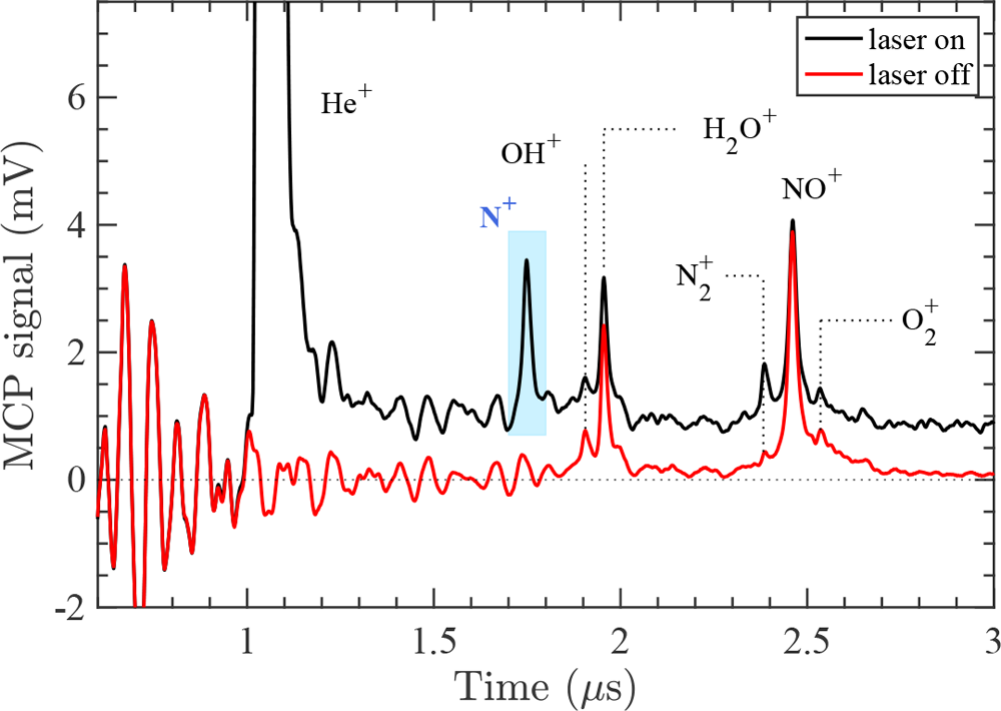
Example TOF mass spectrum for the He(*n*) + NO reaction, recorded with (black) and without (red) turning the Rydberg-excitation laser on. The He atoms were excited to the (*n*, *k*, *m*) = (30, 21, 0) Rydberg–Stark state, and *v*_Ryd_ was set to 860 m/s. The pale-blue rectangle indicates the time window used for the integration of the reaction product ion (N^+^).

A typical TOF mass spectrum of the He^+^ + NO reaction, recorded with an extraction electric field of ∼310 V/cm, is displayed in Figure 2. The He(*n*) atoms were excited to the (*n*, *k*, *m*) = (30, 21, 0) Rydberg–Stark state. The reaction-observation temporal window is determined to be ∼20 μs from the geometric overlap between the He(*n*) atoms and the NO molecules in the detection volume. When the Rydberg-excitation laser is on [black trace in Figure 2], several peaks are clearly visible. The first, most prominent peak (at ∼1.1 μs) originates from field-ionization of the He(*n*) atoms by the extraction pulse. The large intensity of the He^+^ peak [which is cut off in Figure 2a] indicates that only a small fraction (typically less than ∼1%) of the He(*n*) atoms react, as explained in refs (18) and (19). The second peak in Figure 2, with an arrival time of ∼1.75 μs, can be assigned to the N^+^ ions generated in the He^+^ + NO reaction. The other peaks, corresponding to OH^+^, H_2_O^+^, N_2_^+^, NO^+^, and O_2_^+^ ions, originate from Penning-ionization processes involving the metastable He atoms and background water, nitrogen, nitric oxide, and oxygen gases present in the vacuum chamber.

**Figure 3:**
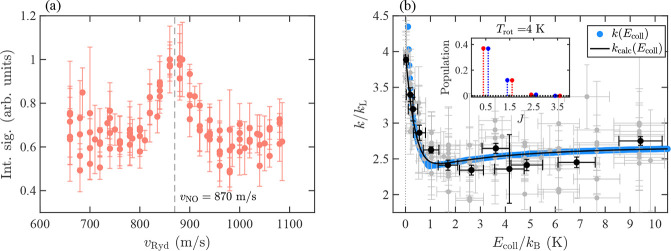
(a) Integrated N^+^ product ions, measured by field ionization of N(*n*) produced in the reaction between NO and He(*n*) atoms excited to the (*n*, *k*, *m*) = (35, 26, 0) Rydberg–Stark state, as a function of the velocity of the He(*n*) atoms (*v*_Ryd_). The vertical dashed line indicates the mean velocity of the NO beam (*v*_NO_ = 870 m/s). (b) Collision-energy dependence of the reaction product-ion yield. The gray and black circles with error bars represent the individual data sets presented in (a) and the averaged and binned data set, respectively. The black line and blue dots are the calculated reaction capture rate coefficients for a rotational temperature of the NO beam of *T*_rot_ = 4 K and Δ*T*_res_ = 100 mK, obtained with and without taking the finite experimental energy resolution into account, respectively. The vertical axis label is for the calculated rate constant, and the experimental data are in arbitrary units because the experiment does not provide absolute rate coefficients. The inset shows the rotational-state occupation probabilities of NO at *T*_rot_ = 4 K. The red and blue dots with corresponding dotted vertical lines refer to the positive- and negative-parity components of the Λ-doublets, respectively.

To determine which peaks correspond to the product ions of the reaction between the He(*n*) atoms and NO molecules, we also record a mass spectrum with the Rydberg excitation laser turned off [red trace in Figure 2]. In this TOF mass spectrum, we can identify all but the He^+^ and N^+^ ions, with intensities that are the same as those obtained with the Rydberg-excitation laser turned on. These TOF spectra indicate that the N^+^ ion is the only product ion of the He^+^ + NO reaction under our experimental conditions, in line with earlier studies of this reaction.^54−57^ The background signal observed beyond 1 μs in the black trace of Figure 3 comes from the slow ionization of the He(*n*) Rydberg atoms caused by blackbody-radiation-induced transitions and tunneling ionization in the extraction electric field.

Because of its spectator role, the Rydberg electron is expected to remain attached to the N^+^ product ion without significant change of the principal quantum number *n*, as demonstrated earlier for the reaction between He(*n*) atoms and CO molecules.^50^ We have verified experimentally that the Rydberg electron also only acts as a spectator in the present case (see Supporting Information).

Figure 3 displays the yield of the N^+^ reaction product ion, obtained after exciting the He atoms to the (*n*, *k*, *m*) = (35, 26, 0) Rydberg–Stark state, as a function of *v*_Ryd_ [(a), red points] and *E*_coll_ [(b), gray points]. It presents data sets accumulated over several days and under the same experimental conditions. Each data point (vertical error bar) represents the average (standard deviation) of the integrated N^+^ signal from five consecutively recorded TOF mass spectra, each representing an average over 500 experimental cycles. The vertical dashed line in (a) indicates the mean velocity (870 m/s) of the NO beam.

The five data sets were averaged and binned according to the collision energy [black circles in Figure 3b], with bin sizes chosen to reflect the experimental energy resolution Δ*E*_coll_ [see eq 3] and represented by the horizontal error bars. The averaged data show no significant dependence on *E*_coll_/*k*_B_ between ∼10 and ∼1.5 K. However, below ∼1 K, a striking increase of the reaction yield can be observed, with a pronounced maximum at *E*_coll_ = 0. This sharp enhancement near zero collision energy represents the most remarkable result of the present investigation and differs markedly from the behavior observed under similar experimental conditions for the He^+^ + CO → He + C^+^ + O reaction^50,59^ although NO and CO have similar molecular structures and electric dipole moments (−0.1574(14) D^60,61^ vs 0.112 D,^62^ respectively). In the case of the He^+^ + CO reaction, the rate coefficient was found to *decrease* by about 30% at collision energies below ∼ *k*_B_·5 K, which was interpreted as arising from the negative value of the quadrupole moment of CO (*Q*_*zz*_ = −2.839 D Å)^63^ on the basis of rotationally adiabatic channel calculations.^50,59^

## 4 Calculations of the Rate Coefficients

To understand the origin of the different behaviors observed for the He^+^ + CO and He^+^ + NO reactions, the capture rate coefficient of the He^+^ + NO reaction was calculated using the same rotational-adiabatic-channel model we used to analyze the results obtained for the He^+^ + CO reaction. In this model, inspired by the earlier work of Clary and co-workers^15,21,22^ and Troe and co-workers,^23,64^ the long-range interaction
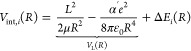
4is determined as the sum of the Langevin interaction potential *V*_L_(*R*) and the rotational-state-specific Stark shift Δ*E*_*i*_(*R*) of NO in the electric field of the He^+^ ion at the ion–molecule separation *R*. In eq 4, *L* is the angular momentum of the colliding ion–molecule system , μ is the reduced mass, α′ is the average polarizability volume of the neutral molecule [α′(NO) = 1.698 × 10^–30^ m^3^],^65^ and the index *i* = (*J*Ω*Mp*) labels the rotational state of the NO molecule in zero electric field (see below).

The Stark shifts Δ*E*_*i*_(*R*) are the eigenvalues of the sum of the rotational Hamiltonian^66^ and the charge-dipole (λ = 1) and charge-quadrupole (λ = 2) interaction matrices  and , respectively, with matrix elements^17,50^
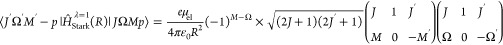
5and
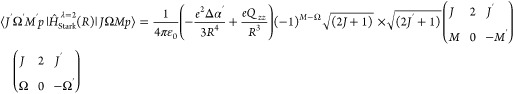
6In these equations, μ_el_ is the permanent electric-dipole moment of NO, ϵ_0_ is the permittivity of free space, *Q*_*zz*_ represents the component of the rank-two, traceless quadrupole-moment tensor describing the quadrupole of NO in the molecular center-of-mass reference frame, Δα′ accounts for the anisotropic part of the charge–induced-dipole interaction, and *p* designates the parity of the rotational states. The charge-dipole interaction mixes states of opposite parity.

**Figure 4:**
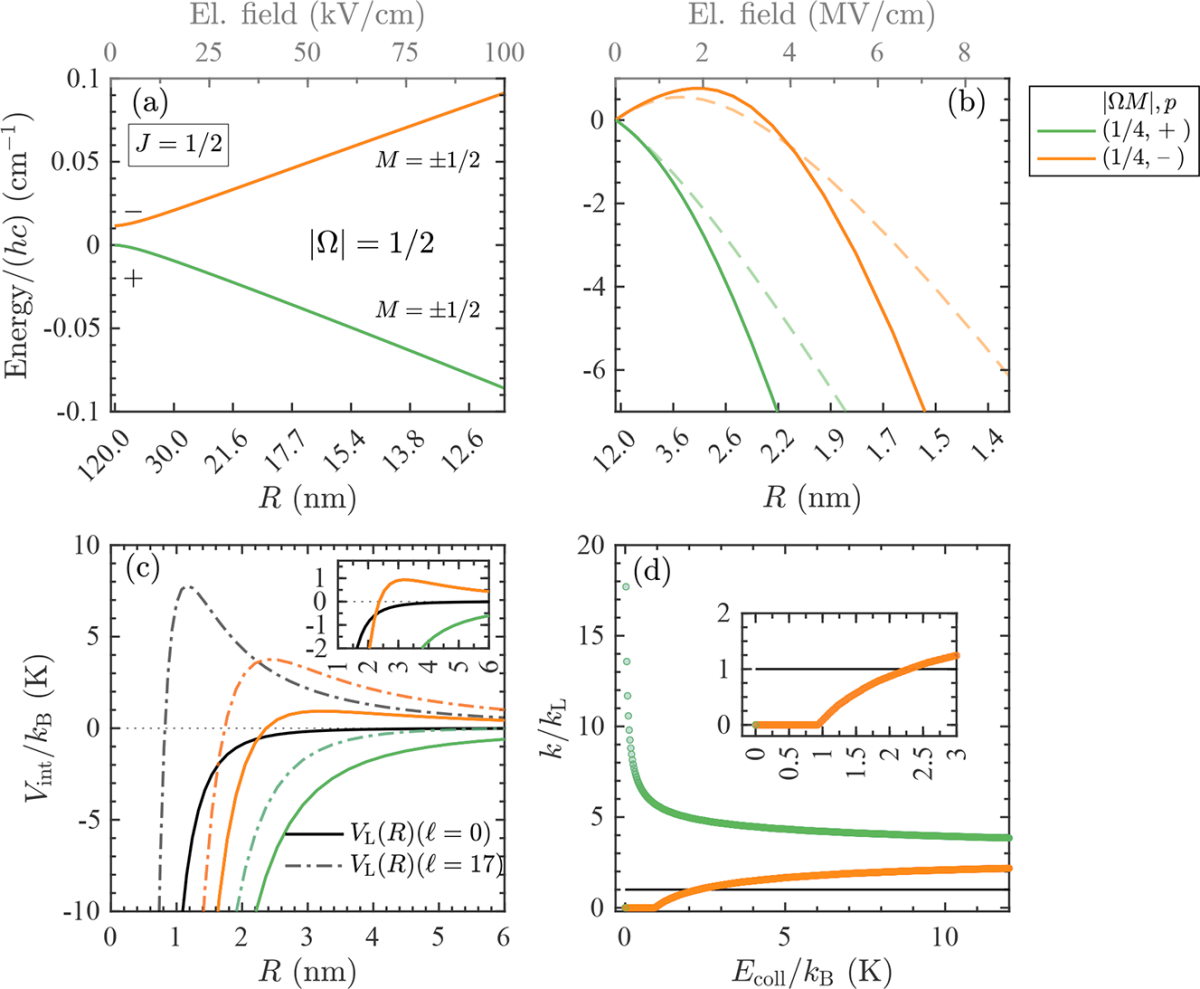
(a, b) Calculated Stark shifts of the X ^2^Π_1/2_, *J* = 1/2 rotational states of NO in the field of the ion. The dashed lines show the ion-dipole Stark effect only. The Stark states are labeled according to the product |Ω*M*| and the parity of the rotational level in zero field. (c) Total interaction potentials for the states in (a) and (b) for a collision with  () in solid (dash-dotted) colored lines, together with the Langevin interaction potential in black (solid and dash-dotted lines, respectively). (d) Calculated rotational-state-dependent capture rate coefficients, normalized to the Langevin rate constant *k*_L_.

**Figure 5:**
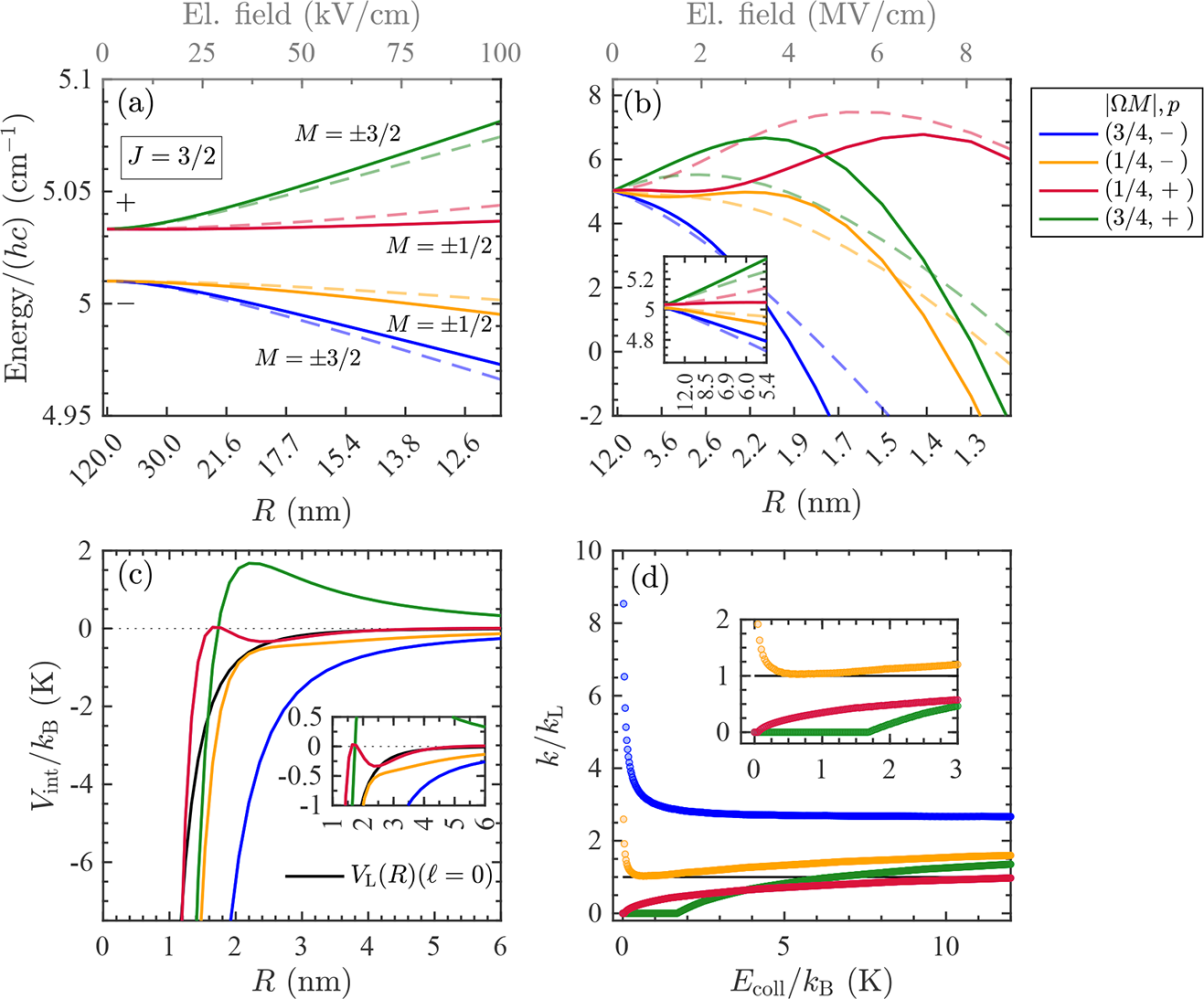
(a, b) Calculated Stark shifts of the X ^2^Π_1/2_, *J* = 3/2 rotational states of NO in the field of the ion. The dashed lines show the ion-dipole Stark effect only. The Stark states are labeled according to the product |Ω*M*| and the parity of the rotational levels in zero field. (c) Total interaction potentials for the states in (a) and (b) for a collision with  (colored lines), together with the Langevin interaction potential (black). (d) Calculated rotational-state-dependent capture rate coefficients, normalized to the Langevin rate constant *k*_L_.

NO is a free radical with open-shell electronic configuration  and a  ground state, where , and Λ and Σ are the quantum numbers associated with the projections of the electronic orbital and spin angular momenta, respectively, on the NO internuclear axis. In the X ^2^Π ground state, the NO rotational levels at zero field are well described by Hund’s angular-momentum coupling case (a)^66,67^ with wave functions labeled |*J*Ω*Mp*⟩. At the low temperature of our supersonic beam, only the lowest two rotational levels (*J* = 1/2, 3/2) of the lower spin–orbit component (^2^Π_1/2_) are significantly populated [see Figure 3b]. Their energy structure is depicted in Figures 4 and 5. Each rotational level is a Λ-doublet comprising two states of opposite parity (*p* = ± 1), the energetic order of which alternates with successive *J* values.

The electric dipole (μ_el_) and quadrupole (*Q*_*zz*_) moments of the X ^2^Π_1/2_(*v* = 0) ground vibronic state of NO are −0.1574(14) D^60,61^ and −2.421 D Å,^25^ respectively. To calculate the state-specific Stark shifts Δ*E*_*i*_(*R*), we expressed the sum of the rotational Hamiltonian and the charge-dipole and charge-quadrupole interactions  (see eqs 5 and 6, respectively) in matrix form in a Hund’s case (a) basis with *J*_max_ = 11.5 and determined its eigenvalues for ion–molecule separations *R* in the range from 120 nm (corresponding to an electric field of 1 kV/cm) down to 0.1 Å (corresponding to 1.4 × 10^8^ kV/cm). The Stark shifts of states with |Ω| = 1/2 and *J* = 1/2 and 3/2 are depicted in Figure 4a,b and Figure 5a,b, where the lower and upper axes represent the intermolecular distance and the electric field, respectively.

The charge-dipole interaction  couples levels of opposite parity, which leads to a close-to-linear Stark effect at ion–molecule distances *R* ≲ 50 nm, corresponding to an electric field of ≳5.8 kV/cm [see Figure 4a and Figure 5a]. The Stark shifts remain approximately linear in the field strength down to about *R* = 5 nm (up to *F* = 0.5 MV/cm) [see Figure 4b and the inset of Figure 5b]. They are proportional to |Ω*M*|, the upper (lower) Λ-doubling components being low-field (high-field)-seeking. Below *R* ≈ 4 nm, corresponding to electric fields *F* ≳ 1 MV/cm and field gradients |*∂F*/*∂R*|≳ 5.2 × 10^20^ V/cm^2^, the Stark shifts are no longer linear. At even shorter distances, all Stark states become high-field-seeking because of the field-induced coupling with higher-lying rotational levels.

The dashed lines in Figure 4b and Figure 5a,b correspond to calculations neglecting the effects of the charge-quadrupole interaction. The comparison with the Stark shifts obtained when including both the charge-dipole and charge-quadrupole interactions (full lines) indicates that the charge-quadrupole interaction only plays a minor role in the He^+^ + NO → He + N^+^ + O reaction. This observation differs from what is found in the case of the He^+^ + CO → He + C^+^ + O reaction (see below).

Figures 4c and 5c compare the state-specific interaction potentials *V*_int,*i*_(*R*) [see eq 4] for the states displayed in (a) to the Langevin interaction potential, *V*_L_(*R*). The solid lines depict *V*_int,*i*_(*R*) and *V*_L_(*R*) for a head-on collision . To illustrate the effect of including the Stark shifts Δ*E*_*i*_(*R*) on the centrifugal potential energy barriers of the colliding ion–molecule pair, Figure 4c also shows the respective interaction potentials for a collision with  (dash-dotted lines).  was chosen because it is well-suited to illustrate the effects of the centrifugal barrier. The high-field-seeking states associated with the lower Λ-doubling components [(|Ω*M*|, *p*) = (1/4, + ) for *J* = 1/2, and (3/4, – ) and (1/4, – ) for *J* = 3/2] have interaction potentials that are more attractive than *V*_L_(*R*), implying an enhancement of the rate coefficients at low collision energies compared to the Langevin rate coefficient *k*_L_. In contrast, the low-field-seeking states all have a potential barrier, even for , which implies that their rate coefficients vanish near *E*_coll_ = 0.

The state-specific rate coefficients were obtained using the equation
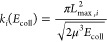
7where *L*_max,*i*_ is the maximal angular momentum fulfilling the condition *V*_int,*i*_(*R*) ≤ *E*_coll_. The results are presented in Figures 4d and 5d. Whereas the rate coefficients corresponding to low-field-seeking states vanish at the lowest collision energies (see insets), as expected, those corresponding to high-field-seeking Stark states increase rapidly below ∼*k*_B_·1 K, reaching values of more than 18 *k*_L_(9 *k*_L_) at *E*_coll_/*k*_B_ = 25 mK for states with *J* = 1/2 (*J* = 3/2). In our previous studies, we found such rate enhancements at low collision energies to be characteristic of reactions between ions and strongly polar molecules, such as NH_3_^18^ (μ_el_(NH_3_) = 1.47 D)^68^ and CH_3_F^17^ (μ_el_(CH_3_F) = 1.86 D).^69^ The dipole moment of NO is, however, an order of magnitude weaker than in these molecules. Our calculations reveal that the effect of the dipole moment in NO is enhanced by its open-shell structure and the near degeneracy of rotational levels of opposite parity resulting from the Λ-doubling.

The total *E*_coll_-dependent capture rate coefficients corresponding to our measurements are obtained as sums of the rotational-state-dependent rate coefficients, weighted by the occupation probability of the (*J*Ω*Mp*) levels in NO at the rotational temperature *T*_rot_ of the supersonic beam, according to the equation
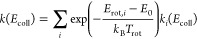
8where *E*_0_ is the energy of the ground (*J*Ω*Mp*) = (1/2, 1/2, 1/2, +) state. To compare with the experimental data, we determine *k*_calc_(*E*_coll_) from *k*(*E*_coll_) by performing an average over the distribution of collision energies of the NO and He(*n*) reactants in the merged beams. This distribution is given in good approximation by a near-thermal velocity distribution, with width Δ*E*_coll_ (see eq 3), primarily limited by the distribution Δ*E*_coll_(*E*_coll_ = 0) = *k*_B_Δ*T*_res_. To reach the best agreement between *k*_calc_ and the experimental data, we vary the values of *T*_rot_ and Δ*T*_res_. The best fit parameters were found to be *T*_rot_ = 4.0(0.5) K and Δ*T*_res_ = 100(25) mK, consistent with previous measurements with our setup.^17−20^

The occupation probabilities of the rotational states of NO at the temperature *T*_rot_ = 4 K are depicted in the inset of Figure 3b, where the red (blue) points and vertical dashed lines correspond to the Λ-doubling components of positive (negative) parity. The pale blue dots in Figure 3b are the rate coefficients *k*(*E*_coll_) averaged over the rotational states populated in the experiment according to eq 8 after normalization to the Langevin rate constant, *k*_L_ = 1.624 × 10^–15^ m^3^/s, and the black lines are the capture rate coefficients *k*_calc_(*E*_coll_) obtained by averaging *k*(*E*_coll_) over the collision-energy distribution, as explained in ref (17). The good agreement between the experimental and calculated data validates the approach followed to calculate the capture rate coefficients. In particular, the enhancement of the rate coefficients observed at the lowest collision energies is quantitatively accounted for by the calculations. One should note that capture rate coefficients do not include the effects of the charge transfer between He^+^ and NO at short-range. It is conceivable that not all capture processes lead to charge transfer. The excellent agreement between the measured and calculated collision-energy dependence of the rate coefficients, however, indicates that the yield of the charge-transfer reaction does not depend on the collision energy over the (narrow) range of collision energies investigated here. Nevertheless, calculations of the charge-transfer cross sections to the energetically accessible states of NO^+^ and of their predissociation would be needed for comparison with the present results.

The overall behavior of the rate coefficients differs from what was observed for the He^+^ + CO → He + C^+^ + O reaction. In that reaction, the effects of the charge-quadrupole interaction were found to be dominant over those of the charge-dipole interaction (compare Figures 4 and 5 with Figure 4 of ref (59)). Moreover, the negative sign of the CO quadrupole moment (*Q*_*zz*_ = −2.839 D Å)^63^ was found to be crucial to explain the ∼30% reduction of the rate coefficients at collision energies below ∼*k*_B_·5 K. In the case of the He^+^ + NO → He + N^+^ + O reaction, changing the sign of the quadrupole moment does not significantly affect the behavior of the rate coefficients below 1.5 K, where the behavior is dominated by the effects of the charge-dipole interaction. However, it affects the slopes of the rate coefficients in the region between 2 and 10 K. For negative values of the quadrupole moment, the rate coefficient decreases slightly with decreasing collision in this range (see Figure 3) whereas it slightly increases when the sign of the quadrupole moment is reversed.

## 5 Comparison with Earlier Theoretical Results

Using the rotational-state-specific capture rate coefficients presented above, we can also calculate the thermal rate coefficients *k*(*T*) of the He^+^ + NO reaction, accounting for the different rotational-state population at each temperature, as described in ref (18). Consequently, our results can be used to assess approximations in the determination of rate coefficients at low temperatures, such as the locked-dipole (LD) and the average-dipole-orientation (ADO) approximations.^11,13^ Both approximations are widely used to predict reaction rate coefficients for reactions between ions and polar molecules, in lieu of more sophisticated methods.

**Figure 6:**
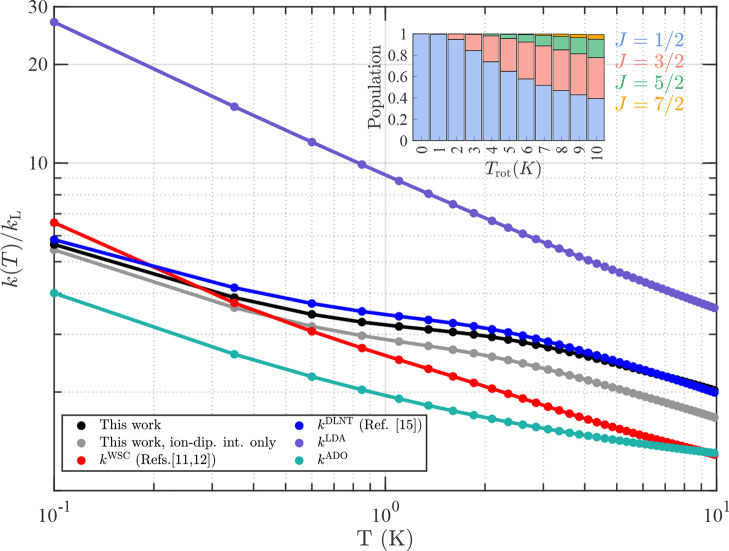
Thermal rate coefficients, normalized to the Langevin rate constant, for a reaction between an ion and an NO molecule. The thermal rate coefficients calculated using the LD and ADO approximations and the analytic expression for the rotational-state-specific rates derived by Wickham, Stoecklin, and Clary^21^ are displayed in purple (*k*^LDA^), green (*k*^ADO^), and red (*k*^WSC^), respectively. The thermal rate coefficients derived from the state-specific rate coefficients reported in ref (25) (*k*^DLNT^) are depicted in blue, and the thermal rate coefficients calculated in the present work including the effects of the ion-dipole and ion-quadrupole interactions, and the ion-dipole interaction only, are drawn in black and gray, respectively. The inset shows the occupation probabilities of the different rotational levels at different temperatures.

Such an assessment for the temperature range from 0 to 10 K is presented in Figure 6. The rate coefficients *k*(*T*) determined in the present work are shown in black and are compared with the thermal rate coefficients calculated using the LD approximation (*k*^LDA^, purple dots and lines) and the ADO approximation (*k*^ADO^, green dots and lines). In the LD approximation, the molecular dipole is considered to be “locked” with the energetically favorable dipole orientation along the collision axis. This dipole orientation leads to a strongly attractive interaction potential and to rate coefficients that increase with decreasing temperature and reach values many times larger than *k*_L_ near 0 K. The LD rate coefficients are always larger than the actual reaction rate coefficients and diverge at zero temperature. In the ADO approximation, the degree of locking between the polar molecule and the ion is assumed to have an average value during the collision and is described by a parameter *c* (0 < *c* < 1). The degree of locking is determined by the ratio of the molecular dipole moment to the molecular polarizability volume.^11^ The value of 0.12 for the *c* parameter used to calculate the low-temperature thermal rate coefficient of the He^+^ + NO reaction in the ADO approximation was determined by extrapolating the values presented in ref (70) to the low-temperature regime. At the low temperatures (*T* < 10 K) studied here, the results of both LDA and ADO treatments deviate significantly from our experimental results and calculations, and the comparison suggests that these approximations should be used with caution when predicting ion–radical reaction rate coefficients at low temperatures, especially when the rotational-level structure of the radical consists of pairs of near-degenerate levels of opposite parity. Whereas the LD approximation leads to a large overestimation of *k*(*T*), the ADO approximation with *c* = 0.12 underestimates *k*(*T*) at low temperatures.

There has been long-standing theoretical interest in the impact of Λ-doubling-mediated first-order Stark shifts in open-shell molecules on the capture rate coefficients of ion–molecule reactions at low temperatures.^21−25^ Wickham et al.^21,22^ were the first to derive rotational-state-specific capture rate coefficients for reactions between an ion and an open-shell ^2^Π linear dipolar molecule. They also derived thermal rate coefficients at low temperatures by retaining only the attractive state-specific potential functions (i.e., those with negative Stark shifts). As they pointed out, this approximation is expected to be more accurate for molecules with a large dipole moment, such as OH (μ_el_(OH) = 1.66 D),^71^ than for molecules such as NO. Nevertheless, their results (*k*^WSC^, red dots and lines in Figure 6) agree with our results to within ∼20% around *T* = 1 K. The main difference with our calculations is an expected faster decrease of their thermal rate coefficient beyond ∼1 K, which originates from their neglecting the contributions from low-field-seeking Stark states to the thermal rate coefficients. Our results indeed indicate that these states contribute to the thermal rate coefficients as soon as *k*_B_*T* becomes larger than the centrifugal barrier for  in the state-specific potentials, see, e.g., the insets of Figures 4c and 5c.

Capture rate coefficients for ion–molecule reactions involving NO have also been calculated by Dashevskaya et al.^25^ and Auzinsh et al.^23,24^ using an adiabatic-channel model from which our own calculations were inspired. The results of these calculations (*k*^DLNT^, blue dots and lines in Figure 6, based on Figures 3, 5, and 6 of ref (25)) are in close agreement with our calculated results, but with values less than ∼10% higher in the range between 0 and 10 K. This difference is likely to be the result of the slightly different value of the dipole moment used, and the inclusion in ref (25) of the angular momentum of NO in the description of the centrifugal repulsion term through their eq 4.

## 6 Conclusions

We have reported, with the example of the He^+^ + NO reaction, the first measurement of the collision-energy-dependent reaction rate coefficient of a reaction between an ion and an open-shell molecule, in the collision-energy range between ∼0 and ∼*k*_B_·10 K. In order to reach such low energies, the He^+^ ion was replaced by a helium atom in a Rydberg state. To be able to both reach near-zero temperature and tune the collision energy, a merged-beam technique was employed, in which the velocity of the Rydberg He atoms was varied using a surface-electrode Rydberg–Stark decelerator and deflector.^53^ We observed N^+^ + O + He as the sole product channel and detected a large enhancement of the reaction yield at collision energies below ≲*k*_B_·1.5 K. We interpreted our experimental results through calculations of the rotational-state-specific capture rate coefficients based on a rotationally adiabatic channel model inspired by earlier theoretical work.^16,72^ The observed enhancement of the reaction yield at low collision energies is attributed to the linear Stark shifts of the near-degenerate Λ-doubling components of opposite parity of the rotational energy levels in NO in the electric field of the colliding ion. The interaction between the opposite-parity rotational energy levels of NO leads to linear Stark shifts at relatively low electric fields. This effect lowers the energies of the lower Λ-doubling components to a much higher degree than would be expected for a closed-shell molecule with a small dipole moment. Attractive long-range interaction potentials result, which leads to a strong increase of the rate coefficients at low collision energies.

The collision-energy dependence of the reaction yield of the He^+^ + NO reaction (sharp increase near 0 K) markedly differs from the behavior observed in the He^+^ + CO reaction (a decrease of ∼30% near 0 K),^50,59^ although NO and CO have similar masses, polarizabilities, rotational constants, and electric dipole and quadrupole moments. The presence of the Λ-doubling in NO effectively enhances the effect of the molecular electric dipole moment and makes the ion-dipole interaction the dominant long-range interaction, in contrast to the case of CO where the ion-quadrupole was found to be the dominant long-range interaction.^59^ Our experimental results have verified theoretical predictions made by Clary and his co-workers more than 30 years ago and responded, with some delay, to their encouragement and recommendation to experimentalists that “particular emphasis should be placed on the variation of the rate coefficient with temperature close to 0 K”.^21^

## Supporting Information Available

Test of the spectator role of the Rydberg electron: field-ionization measurements of the He(*n*) Rydberg-atom reactant and of the N(*n*′) Rydberg-atom product demonstrating that the Rydberg electron is not affected by the ion–molecule reaction taking place within its orbit, i.e., *n* ≈ *n*′, where *n* and *n*′ are the respective principal quantum numbers (PDF)
