# Supplementary material for: Reaction of an Ion and a Free Radical near 0 K: He+ + NO → He + N+ + O
Source: J Phys Chem A. 2023 Feb 8;127(6):1458–68. doi: 10.1021/acs.jpca.2c08221 (PMC9940198; doi:10.1021/acs.jpca.2c08221)
Supplement: Supplementary file 1 — jp2c08221_si_001.pdf [file jp2c08221_si_001.pdf]

Reaction of an ion and a free radical near 0 K: He^+ + NO$\rightarrow$ He + N^+ + O

Valentina Zhelyazkova, Fernanda B. V. Martins, Serena Schilling, and Frédéric Merkt*

Laboratory of Physical Chemistry, ETH Zürich, CH-8093 Zürich

E-mail: frederic.merkt@phys.chem.ethz.ch

Supplementary Material: The spectator role of the Rydberg electron

To verify that the Rydberg electron only plays a spectator role in the He(n) + NO reaction, we performed a set of measurements in which the strength of the extraction field was gradually increased, up to the maximum electric field of $\sim$ 484 V/cm that can be applied across the extraction region in our setup while maintaining a sufficient mass resolution. The results of such a set of measurements, in which the He atoms were excited to the $(n, k, m) = (45, 36, 0)$ Rydberg-Stark state, are displayed in Fig. 1(a). As the extraction electric field (F_{extract}) is increased from 44 V/cm to 484 V/cm in steps of 44 V/cm, the ion flight times are reduced, as expected. The intensities of the ions generated by Penning-ionization processes (e.g., OH^+, H_2O^+, N_2^+, NO^+ and O_2^+) increase only slightly with increasing extraction electric-field strength, reflecting the increase of the detection efficiency of faster ions by the MCP detector, as discussed in Ref. 1. The peak corresponding to the N^+ ions is highlighted by the pale blue line. Its intensity increases faster with F_{extract} than in the case of the Penning-ionization product ions, indicating that the reaction product is created in a Rydberg state [N(n)] which is more efficiently ionized with increasing electric field.

The integrated signal of the detected N^+ ions is displayed as a function of F_{extract} as black dots in Fig. 1(b), after correcting for the field-dependent detection efficiency and scaling up by a factor of 100. The top and bottom panels correspond to measurements performed with the He atoms excited to the Rydberg-Stark states $(n, k, m) = (45, 36, 0)$ and $(40, 31, 0)$, respectively. The open circles depict the pulsed-field-ionization (PFI) signals of the He(n) atoms, recorded in a separate measurement without the NO beam present and normalized to their maximal intensity. Higher extraction electric fields could be used to detect He(n) by PFI (up to $\sim$ 1214 V/cm), because the Wiley-McLaren condition for high mass resolution did not have to be satisfied. The slew rate of the applied extraction fields ($\mathrm{d}F_{\text{extract}}/\mathrm{d}t \gtrsim 1.43 \times 10^9$ $\mathrm{V\,cm^{-1}\,s^{-1}}$) ensures the diabatic field ionization of the He(n) Rydberg states of helium with $n \geq 35$, as demonstrated in Ref. 1. For both the $n = 40$ and 45 data sets, the He^+ signal

gradually increases with F_{extract} and reaches a plateau at $F_{\mathrm{extract}} \gtrsim 550$ V/cm (350 V/cm) for $n = 40$ ($n = 45$), indicating that complete field ionization of the sample has been reached. The red step functions in Fig. 1(b) represent the diabatic ionization thresholds[2] of the initial $n = 40$ and $n = 45$ states. The positions of the steps roughly coincide with the inflection points of the experimental He(n) PFI profiles.

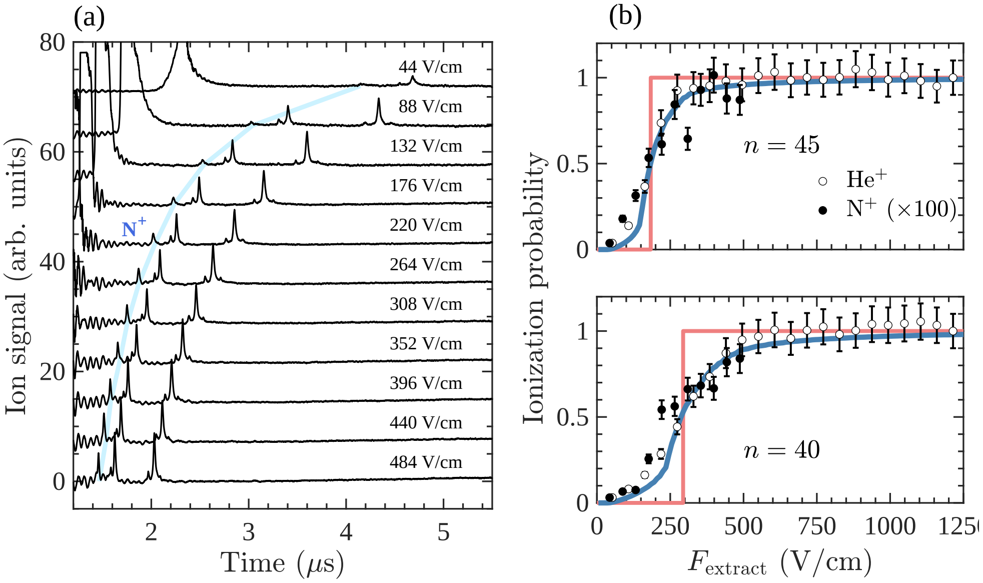

Figure 1: (a) TOF mass spectra recorded with the He atoms initially excited to the $(45, 36, 0)$ Rydberg-Stark state for several extraction fields in the range between 44 to 484 V/cm. The pale-blue line highlights the N$^+$ product ion. (b) Normalized integrated He$^+$ (white circles) and N$^+$ (black circles) ion signals generated by the pulsed field ionization of He(n) and N(n), respectively. The top and bottom panels display the results obtained with the He atoms initially excited to the $(45, 36, 0)$ and $(40, 31, 0)$ Rydberg-Stark states, respectively. The red step function indicates the diabatic field ionization threshold for the $n = 45$ and $n = 40$ states, and the blue curve displays the diabatic ionization probability corresponding to the distributions of n states predicted by Monte-Carlo simulations.

The observed rise of the field-ionization signal with increasing field is, however, less sharp than predicted by the red step functions in Fig. 1(b), indicating a redistribution of the population of initially prepared Stark states to neighboring Rydberg states. Three distinct mechanisms contribute to this redistribution: (i) a randomization of the value of k of the He(n) atoms in the zero-field region between the deflector and the reaction zone; (ii) radiative transitions between Rydberg states, primarily those induced by blackbody radiation, during

the ~ 100 μs-long flight time between the photoexcitation spot and the reaction zone; and (iii) the reaction He(n) + NO $\rightarrow$ He + N(n') + O. To quantify the role of mechanisms (i) and (ii), we use a Monte-Carlo simulation program described in Refs. 1,3, with which we determine the distribution of the He Rydberg atoms over the (n, k, m) states in the reaction zone and their field-ionization yield following the procedure outlined in Ref. 1. These yields are depicted as blue lines in Fig. 1(b) and closely reproduce the experimentally observed field-ionization yields. This analysis leads to the conclusion that the reaction [mechanism (iii)] does not lead to significant changes of n values, i.e., $|n' - n| \leq 1$, which confirms that the Rydberg electron is a spectator to the reaction. These conclusions are identical to those drawn in our previous study of the He(n) + CO $\rightarrow$ He + C(n') + O reaction.[1]

References

(1) Martins, F. B. V.; Zhelyazkova, V.; Seiler, Ch..; Merkt, F. Cold ion chemistry within a Rydberg-electron orbit: Test of the spectator role of the Rydberg electron in the He(n) + CO $\rightarrow$ C(n') + O + He reaction. *New J. Phys.* **2021**, *23*, 095011.

(2) Gallagher, T. F. *Rydberg Atoms*; Cambridge University Press, 1994.

(3) Seiler, Ch.; Agner, J. A.; Pillet, P.; Merkt, F. Radiative and collisional processes in translationally cold samples of hydrogen Rydberg atoms studied in an electrostatic trap. *J. Phys. B: At. Mol. Opt. Phys.* **2016**, *49*, 094006

.
